# Joint Detection and DOA Tracking with a Bernoulli Filter Based on Information Theoretic Criteria

**DOI:** 10.3390/s18103473

**Published:** 2018-10-15

**Authors:** Guangpu Zhang, Ce Zheng, Sibo Sun, Guolong Liang, Yifeng Zhang

**Affiliations:** 1 Acoustic Science and Technology Laboratory, Harbin Engineering University, Harbin 150001, China; zhangguangpu@hrbeu.edu.cn (G.Z.); zhengce_uae@hrbeu.edu.cn (C.Z.); liangguolong@hrbeu.edu.cn (G.L.); zhangyifeng1@hrbeu.edu.cn (Y.Z.); 2 Key Laboratory of Marine Information Acquisition and Security (Harbin Engineering University), Ministry of Industry and Information Technology, Harbin 150001, China; 3 College of Underwater Acoustic Engineering, Harbin Engineering University, Harbin 150001, China; 4 Qindao Haina Underwater Information Technology Co., Ltd., Qindao 266500, China

**Keywords:** DOA, Bernoulli filter, track before detect

## Abstract

In this paper, we study the problem of the joint detection and direction-of-arrival (DOA) tracking of a single moving source which can randomly appear or disappear from the surveillance volume. Firstly, the Bernoulli random finite set (RFS) is employed to characterize the randomness of the state process, i.e., the dynamics of the source motion and the source appearance. To increase the performance of the detection and DOA tracking in low signal-to-noise ratio (SNR) scenarios, the measurements are obtained directly from an array of sensors and allow multiple snapshots. A track-before-detect (TBD) Bernoulli filter is proposed for tracking a randomly on/off switching single dynamic system. Secondly, since the variances of the stochastic signal and measurement noise are unknown in practical applications, these nuisance parameters are marginalized by defining an uninformative prior, and the likelihood function is compensated by using the information theoretic criteria. The simulation results demonstrate the performance of the filter.

## 1. Introduction

Detection and direction-of-arrival (DOA) estimation using an array of sensors are important topics in signal processing and have many applications, such as in radar, sonar, communications and acoustics [1,2,3,4,5,6,7]. Considering moving sources in a noisy environment [1,2], it is desirable to use both the spatial and temporal information for better performance, since the DOAs between consecutive time steps are highly correlated. In [7,8,9,10], recursive Bayesian approaches are proposed for tracking the DOA of moving sources. However, most existing DOA tracking schemes assume that the number of sources is known and fixed. This assumption is often violated in many practical situations, since a source can appear or disappear from the surveillance volume during the observation interval. Considering an unknown and time-varying number of sources, two frameworks have emerged for tracking: track-after-detect (TAD) and track-before-detect (TBD). 

In a TAD system, this task is always decomposed into separated modules. A pre-processing module is applied to obtain a set of detected points. Afterwards, a multitarget tracking (MTT) procedure is conducted on these detected points. These detected points are generated by the thresholding stage, which could result in a loss of information. The performance degrades dramatically under a low signal-to-noise ratio (SNR) or a small number of snapshots [11]. For the pre-processing module, there have been several methods developed for detecting and localizing sources using an array of sensors. The conventional beamforming method and the minimum-variance-distortionless-response (MVDR) method [12], which rely on picking peaks above a selected threshold, are the most widely used. Maximum likelihood based DOA estimation algorithms are investigated in [13] coincide with Akaike information criterion (AIC) and the minimum description length (MDL) for detection. The Bayesian predictive densities (BPD) approach was developed in [14] using a prior probability density function. Further, high-resolution subspace based methods have been studied in [15,16] with an unknown number of sources. 

A TBD system tracks targets directly using the raw data, without a pre-processing module. Indeed, any detection is conducted after tracking. More information is reserved, which allows better performance in the challenging environment. A TBD particle filter is firstly proposed in [10] for DOA tracking, coupled with a reversible jump Monte Carlo Markov chain (RJMCMC) step to handle fluctuations in the source number. This method can be only carried out using a single snapshot. Hence, it is sensitive to incorrect model order initialization and degrades heavily when it comes to noise [17].

Recently, Mahler introduced the concept of random finite sets (RFS) to handle uncertainty in the number of targets for tracking [18]. Since the implementation of the RFS formulation of the optimal Bayes filter for multiple dynamic systems is computationally very demanding, some moment-based approximations have emerged recently for multisource DOA tracking: the probability hypothesis density (PHD) filter and cardinalized PHD (CPHD) filter. In [17], a PHD filter is investigated using the separable observations. A CPHD filter is investigated in [19] with known variances of signal and noise and only allows a single snapshot during recursion. In [20], a multi-Bernoulli filter for DOA tracking is proposed, by using the multiple signal classification (MUSIC) pseudo-spectrum as the likelihood function. 

Usually, it is not known whether the target exists or not in a particular surveillance volume that is of interest. Our aim is to determine, from the measurement, the existence of the target and its state. The Bernoulli filter provides the optimal Bayes filter for a single dynamic system which randomly switches on and off [21]. The main feature of the Bernoulli filter is that the underlying state is treated as a set (which can be empty or singleton) instead of a vector augmented with the binary existence variable. Since it has no analytic solution, particle filter implementation provides a solution to Bernoulli recursions under non-linear/non-Gaussian cases, also known as the Bernoulli particle filter (BPF) [22]. The use of Bernoulli filter for TBD systems can be found in host of applications, such as multiple input multiple output (MIMO) radar [23], acoustic or speech sources [24,25], and similar.

In this paper, we propose a track-before-detect Bernoulli filter (TBD-Ber) for single source detecting and DOA tracking based on information theoretic criteria. The novelty of this work is twofold. Firstly, we consider an array of sensors measurement model, by using the measurements directly obtained from the array elements’ raw data. Since more information is reserved, the performance of detection and DOA tracking is improved in a noisy environment. Furthermore, it has no need of the probability of miss-detect and false alarms, which are hard to obtain in passive radar/sonar applications. Secondly, the variances of the stochastic signal and measurement noise are unknown in practical applications, known as nuisance parameters. Both the signals and noise variances can be marginalized by defining an uninformative prior. Since this is improper and unreliable for detection, we use information theoretic criteria (AIC and MDL) to compensate for the likelihood function, resulting in a penalty term. Simulation experiments are carried out and showcase the performance in challenging environments where the SNR is low and the number of snapshots is small.

The paper is organized as follows. Section 2 introduces the measurement model using an array and the formulation of Bayesian recursion based on Bernoulli RFS. Section 3 presents the derivation of the update equations and the likelihood function based on information theoretic criteria. Particle implementation is also presented. The performance metric and discussions are presented in Section 4. Simulated experiments are organized in Section 5. Finally, conclusions and future works are discussed in Section 6.

## 2. Background

### 2.1. Measurement Model Using an Array of Sensors

Consider an array composed of M sensors with arbitrary locations and arbitrary directional characteristics. Assume that a narrowband signal *s*(*k*), with a known center frequency *ω*_0_, is impinging on the array from DOA θϵ(−90, 90], at discrete time *k.* For simplicity, assume that source and sensors are located in the same plane and that the source is in the far-field of the array. The received signal model for an array at time *k* can be written as

(1)  z(k)=a(θ)s(k)+n(k) 

where **z**(*k*) is a *M* × 1 received signal vector, **n**(*k*) is a *M* × 1 vector of additive noise, *s*(*k*) is a signal for a single source at the reference point, $a\left(\theta \right)={\left[e^{-j\omega_{0}\tau_{1}\left(\theta \right)},\ldots ,e^{-j\omega_{0}\tau_{M}\left(\theta \right)}\right]}^{T}$ is the *M* × 1 manifold vector, $\tau_{m}\left(\theta \right)$ is the propagation delay between the reference point and *m*th sensor to a wave front impinging from direction *θ*. The superscript *T* represents transposition.

For a source located at the far-field that moves relatively slowly, the DOA parameter θ is approximately stable during a small number of snapshots at each time step. At time *k*, *N* snapshots are taken into account and the corresponding DOA is *θ_k_*. The received data at time *k* with previous *N* snapshots can be written by the following matrix notation:

(2) $$\;Z_{k}=\left[z\left(kN+1\right),\ldots ,z\left(kN+N\right)\right]\;$$

where the received data **Z***_k_* is a *M* × *N* matrix. Then, Equation (1) can be written as:

(3) $$\;Z_{k}=a\left(\theta_{k}\right)S_{k}+N_{k}\;$$

where the source signal data is a 1 × *N* vector and additive noise data is *M* × *N* matrix, separately written as:

(4) $$\;S_{k}=\left[s\left(kN+1\right),\ldots ,s\left(kN+N\right)\right]\;$$

(5) $$\;N_{k}=\left[n\left(kN+1\right),\ldots ,n\left(kN+N\right)\right]\;$$

Assume both the signal **S***_k_* and the noise **N***_k_* are independent, identically distributed (i.i.d.), with a zero-mean and complex Gaussian process distributed. The source signal $S_{k}\sim CN\left(0,\sigma_{s}^{2}\right)$ and the noise $N_{k}\sim CN\left(0,\sigma_{n}^{2}I_{M}\right)$ are independent, where **I***_M_* denotes a *M* × *M* identity matrix, $\sigma_{s}^{2}$ and $\sigma_{n}^{2}$ are the real variances of signal and noise, respectively. The distribution of the received signal also follows a zero-mean, complex Gaussian process **Z***_k_*~*CN*(0,**R***_k_*), where the covariance matrix is $R_{k}=\sigma_{s}^{2}a\left(\theta_{k}\right)a^{H}\left(\theta_{k}\right)+\sigma_{n}^{2}I_{M}$. The superscript *H* represents conjugate transposition. Since the true covariance matrix **R***_k_* is not available in practice, the measurement model is the sample-covariance matrix of received signal, given as:

(6) $$\;{\hat{R}}_{k}=\frac{1}{N}Z_{k}Z_{k}^{H}\;$$

### 2.2. Bernoulli RFS Formulation

In practice, the source of interest can enter and exit the surveillance region at random instances. Therefore, it is important to consider techniques that can jointly detect and track a source. Recently, Mahler introduces finite set statistics (FISST) [18] that provides the tool for mathematical representation and a convenient probabilistic model for the representation. The state of a time-varying number of sources can be simply represented by a finite-set valued random variable, also known as a random finite set (RFS). 

The probability density function (PDF) of an RFS $X=\left\{x^{\left(1\right)},\cdots ,x^{\left(n\right)}\right\}$ can be specified by a cardinality distribution ρ(n)=P{|X|=n} and a family of symmetric joint distribution $p_{n}\left(x^{\left(1\right)},\cdots ,x^{\left(n\right)}\right)$, where $n\in {\mathbb{N}}_{0}$ and $x^{\left(1\right)},\cdots ,x^{\left(n\right)}\in \emptyset \cup \chi$ denotes the distribution of its elements over the state space χ, conditioned on cardinality *n*. The PDF of a RFS *X* is defined as

(7) $$f\left(\left\{x^{\left(1\right)},\cdots ,x^{\left(n\right)}\right\}\right)=n!\rho \left(n\right)p_{n}\left(x^{\left(1\right)},\cdots ,x^{\left(n\right)}\right)$$

and its set integral is defined as

(8) $$\;\int f\left(X\right)\delta X=f\left(\emptyset \right)+\overset{\infty }{\underset{n=1}{\sum }}\frac{1}{n!}\int f\left(\left\{x^{\left(1\right)},\cdots ,x^{\left(n\right)}\right\}\right)dx^{\left(1\right)}\ldots dx^{\left(n\right)}\;$$

It is straightforward to show that *f*(*X*) integrates to one (as it should, being a PDF). 

In this paper, the Bernoulli RFS *X* is employed to model the state of a source using a probability *q* to be a singleton distributed according to the ‘spatial’ PDF *s*(**x**) over the state space χ, given as *X* = {*q*, *s*(**x**)}. Thus, the probability of being empty is equal to 1 − *q*. Consider that a source with DOA *θ* is moving with velocity $\dot{\theta }$ (in degree/s), the state vector is constructed by $x=\left[\theta ,\dot{\theta }\right]$. The posterior PDF, at time step *k*, can be represented by a Bernoulli RFS with PDF

(9) $$\;p_{k}\left(X_{k}|z_{1:k}\right)=\{\begin{array}{cc} 1-q_{k}\; & \mathrm{if}\;X_{k}=\emptyset \;\\ q_{k}\cdot s_{k}\left(x_{k}\right) & \mathrm{if}\;X_{k}=\left\{x_{k}\right\}\\ 0 & \mathrm{otherwise} \end{array}\;$$

Considering the dynamics of source presence and absence, the dynamic model $f_{k|k-1}\left(X_{k}|X_{k-1}\right)$ is modeled as a Bernoulli Markov process. Conditional upon $X_{k-1}=\emptyset$, the target can re-enter the scene with probability *p*_R,*k*_ and occupy a kinematic state **x***_k_* with PDF $f_{R,k}\left(x_{k}\right)$, or remain absent from the scene with probability 1 − *p*_R,*k*_. Conditional upon $X_{k-1}=\left\{x_{k-1}\right\}$, the source can survive to the next time step with probability $p_{S,k}\left(x_{k-1}\right)$ and transition to **x***_k_* with PDF $f_{k}\left(x_{k}|x_{k-1}\right)$, or disappear with probability $1-p_{S,k}\left(x_{k-1}\right)$. The dynamic model can be expressed as

(10) $$f_{k|k-1}\left(X_{k}|X_{k-1}\right)=\{\begin{array}{cc} 1-p_{R,k} & \mathrm{if}\;X_{k-1}=\emptyset ,\;X_{k}=\emptyset \\ p_{R,k}\cdot f_{R,k}\left(x_{k}\right) & \mathrm{if}\;X_{k-1}=\emptyset ,\;X_{k}=\left\{x_{k}\right\}\\ 1-p_{S,k}\left(x_{k-1}\right) & \mathrm{if}\;X_{k-1}=\left\{x^{\prime }\right\},\;X_{k}=\emptyset \\ p_{S,k}\left(x_{k-1}\right)\cdot f_{k|k-1}\left(x_{k}|x_{k-1}\right) & \mathrm{if}\;X_{k-1}=\left\{x^{\prime }\right\},\;X_{k}=\left\{x_{k}\right\} \end{array}\;$$

where the $f_{k|k-1}\left(x_{k}|x_{k-1}\right)$ is the traditional transition density when the source survives. In this paper, the constant velocity (CV) model is employed to model the source motion when the source survives and given as

(11) $$\;x_{k}=Fx_{k-1}+Gv_{k}\;$$

where the coefficient matrix **F** and **G** are defined by

(12) $$F=\left[\begin{array}{cc} 1 & \Delta T\\ 0 & 1 \end{array}\right];\;G=\left[\begin{array}{c} \Delta T^{2}/2\\ \Delta T \end{array}\right]$$

where Δ*T* represents the time period in seconds between the previous and current time step, and $v_{k}\sim N\left(0,\sigma_{v}^{2}\right)$ is a zero-mean real Gaussian process used to model the turbulence on the source velocity. Such a constant velocity model has been widely used for DOA tracking problems [7,8,26]. For more complicated trajectories and faster moving sources, a constant acceleration model is used to model the source dynamics [27]. 

It is worthy of mention that the measurements of TBD filters are different from the standard multi-target tracking algorithms (i.e., TAD filters) which are the standard (points) measurements. The non-standard (intensity) measurement **Z***_k_* is a matrix that always exists within a fixed dimension given by (2). Hence, the likelihood function is defined as

(13) $$\;g_{k}\left(Z_{k}|X_{k}\right)=\{\begin{array}{cc} g_{k}\left(Z_{k}|\emptyset \right)\; & \mathrm{if}\;X_{k}=\emptyset \;\\ g_{k}\left(Z_{k}|x_{k}\right) & \mathrm{if}\;X_{k}=\left\{x_{k}\right\}\\ 0 & \mathrm{otherwise} \end{array}\;$$

where $g_{k}\left(Z_{k}|\emptyset \right)$ denotes that the received signal is pure noise. Assuming the posterior PDF of the source state at the time step *k* − 1 is known, this is given as $p_{k-1}(X_{k-1}|Z_{1:k-1})$. The predict and update equations based on Bernoulli RFS modeling become the following

- Prediction
  (14) $$\;p_{k|k-1}\left(X_{k}|Z_{1:k-1}\right)=\int f_{k|k-1}\left(X_{k}|X_{k-1}\right)p_{k-1}(X_{k-1}|Z_{1:k-1})\delta X_{k-1}\;$$
- Update
  (15) $$\;p_{k}\left(X_{k}|Z_{1:k}\right)=\frac{g_{k}\left(Z_{k}|X_{k}\right)p_{k|k-1}\left(X_{k}|Z_{1:k-1}\right)}{\int g_{k}\left(Z_{k}|X\right)p_{k|k-1}\left(X|Z_{1:k-1}\right)\delta X}\;$$

In the predict equation, $f_{k|k-1}\left(X_{k}|X_{k-1}\right)$ is the transition density defined by (10), and $p_{k|k-1}\left(X_{k}|Z_{1:k-1}\right)$ is the prior distribution for the current time step. Consequently, the current distribution of the state can be obtained recursively by using this Bayesian recursion.

## 3. Bernoulli Filtering

### 3.1. Bernoulli Filter

Following from (10), the predicted PDF can also be written in the form of Bernoulli RFS, given as

(16) $$\;p_{k|k-1}\left(X_{k}|Z_{1:k-1}\right)=\{\begin{array}{cc} 1-q_{k|k-1}\; & \mathrm{if}\;X=\emptyset \;\\ q_{k|k-1}\cdot s_{k|k-1}\left(x_{k}\right) & \mathrm{if}\;X=\left\{x_{k}\right\}\\ 0 & \mathrm{otherwise} \end{array}\;$$

where the predicted probability of the existence and predicted ‘spatial’ PDF are:

(17) $$\;q_{k|k-1}=p_{R,k}\cdot \left(1-q_{k-1}\right)+q_{k-1}\cdot p_{S,k}\;$$

(18) $$\;s_{k|k-1}\left(x_{k}\right)=\frac{\left(1-q_{k-1}\right)\cdot p_{R,k}\cdot f_{R,k}\left(x_{k}\right)+p_{S,k}\cdot q_{k-1\;}\cdot \int p_{k|k-1}\left(x_{k}|x\right)\cdot s_{k-1}\left(x\right)dx}{\left(1-q_{k-1}\right)\cdot p_{R,k}+p_{S,k}q_{k-1\;}}\;$$

Following from (15), the posterior probability of the existence and posterior ‘spatial’ PDF of the posterior PDF $p_{k}\left(X_{k}|Z_{1:k}\right)$ are given as

(19) $$\;q_{k}=\frac{q_{k|k-1}\cdot \int g_{k}\left(Z_{k}|x\right)s_{k|k-1}\left(x\right)dx}{\left(1-q_{k|k-1}\right)g_{k}\left(Z_{k}|\emptyset \right)+q_{k|k-1}\int g_{k}\left(Z_{k}|x\right)s_{k|k-1}\left(x\right)dx}\;$$

(20) $$\;s_{k}\left(x\right)=\frac{g_{k}\left(Z_{k}|x_{k}\right)\cdot s_{k|k-1}\left(x_{k}\right)}{\int g_{k}\left(Z_{k}|x\right)s_{k|k-1}\left(x\right)dx}\;$$

### 3.2. Likelihood Function Based on Information Theoretic Criteria

Related to the measurement model, the measurement noise process is assumed to be Gaussian. Conditional upon $X_{k}=\left\{x_{k}\right\},$ the likelihood function can be written as

(21) $$\;g_{k}\left(Z_{k}|x_{k}\right)=\frac{1}{\det{\left(\pi R_{k}\right)}^{N}}\exp\left(-\frac{1}{N}\overset{kN+N}{\underset{i=kN+1}{\sum }}z^{H}\left(i\right)R_{k}^{-1}z\left(i\right)\right)\;$$

If we have the priors of the variances of the source signal and noise, the likelihood function is straightforward. However, these parameters are always unknown in many radar/sonar applications. In order to marginalize these nuisance parameters, the measurement is split into the two complementary subspaces [13,28]. The signal subspace is spanned by the columns of matrix **a**(*θ*), and the orthogonal space is referred to as the noise subspace. According to this decomposition, the measurement vector **z** is the split into two subspace vectors by

(22) $$\;z=G\left(\theta \right)\left[\begin{array}{c} z_{s}\\ z_{n} \end{array}\right]\;$$

where *z_s_* is the source signal subspace scalar and **z***_n_* is the (*M* − 1) × 1 noise subspace vector. Note that $G\left(\theta \right)=\left[u_{s}\left(\theta \right)\;U_{n}\left(\theta \right)\right]$ denotes an *M* × *M* unitary coordinate transformation matrix. According to these definitions we have

(23) $$\begin{array}{cc} P_{a\left(\theta \right)} & =a\left(\theta \right){\left(a^{H}\left(\theta \right)a\left(\theta \right)\right)}^{-1}a^{H}\left(\theta \right)\\ & =u_{s}\left(\theta \right)u_{s}^{H}\left(\theta \right) \end{array}$$

and

(24) $$\;P_{a\left(\theta \right)}^{⊥}=I_{M}-P_{a\left(\theta \right)}=U_{n}\left(\theta \right)U_{n}^{H}\left(\theta \right)\;$$

where $u_{s}\left(\theta \right)$ is the *M* × 1 vector and $U_{n}\left(\theta \right)$ is the *M* × (*M* − 1) matrix, denoting orthogonal vectors that span the source signal and noise subspaces, respectively. $P_{a\left(\theta \right)}$ and $P_{a\left(\theta \right)}^{⊥}$ denote the orthogonal projection on the source signal subspace and noise subspace, respectively. Since the transformation (22) is linear, the total likelihood function can be modified using:

(25) $$\begin{array}{cc} g_{k}\left(Z_{k}|x_{k}\right) & =\frac{g_{k}\left(Z_{s,k}|x_{k}\right)\cdot g_{k}\left(Z_{n,k}|x_{k}\right)}{J\left(z_{s},z_{n};z\right)}\\ & =g_{k}\left(Z_{s,k}|x_{k}\right)\cdot g_{k}\left(Z_{n,k}|x_{k}\right) \end{array}$$

where $Z_{s,k}=\left[z_{s}\left(kN+1\right),\ldots ,z_{s}\left(kN+N\right)\right]$ and $Z_{n,k}=\left[z_{n}\left(kN+1\right),\ldots ,z_{n}\left(kN+N\right)\right]$, $J\left(z_{s},z_{n};z\right)$ is the Jacobian of the transformation Equation (25) and is equal to 1. Since source signal and noise are independent zero mean complex Gaussian processes, their likelihood functions are given as

(26) $$\begin{array}{cc} g_{k}\left(Z_{s,k}|x_{k}\right) & =\frac{1}{{\left|\pi R_{ss}\right|}^{N}}\exp\left(-\frac{1}{R_{ss}}\overset{kN+N}{\underset{i=kN+1}{\sum }}z_{s}\left(i\right)z_{s}^{*}\left(i\right)\right)\\ & =\frac{1}{{\left|\pi R_{ss}\right|}^{N}}\exp\left(-\frac{N{\hat{R}}_{ss}}{R_{ss}}\right) \end{array}$$

and

(27) $$\begin{array}{cc} g_{k}\left(Z_{n,k}|x_{k}\right) & =\frac{1}{{\left(\pi \sigma^{2}I_{M-1}\right)}^{N}}\exp\left(-\frac{1}{\sigma^{2}}\mathrm{tr}\left[\sum_{i=kN+1}^{kN+N}z_{n}\left(i\right)z_{n}^{H}\left(i\right)\right]\right)\\ & =\frac{1}{{\left|\pi \sigma^{2}I_{M-1}\right|}^{N}}\exp\left(-\frac{N\mathrm{tr}\left({\hat{R}}_{nn}\right)}{\sigma^{2}}\right) \end{array}$$

where tr( ) denotes a trace of a matrix, superscript * represents a conjugate, $N{\hat{R}}_{ss}=\sum_{i=kN+1}^{kN+N}z_{s}\left(i\right)z_{s}^{*}\left(i\right)$ and $N{\hat{R}}_{nn}=\sum_{i=kN+1}^{kN+N}z_{n}\left(i\right)z_{n}^{H}\left(i\right)$.

Intuitively, the maximum likelihood estimators of the variances of source signal and noise subspace components are $R_{ss}={\hat{R}}_{ss}$ and $\sigma^{2}={\hat{\sigma }}^{2}=\frac{1}{M-1}\mathrm{tr}\left({\hat{R}}_{nn}\right)$, respectively. If we do not have strong prior beliefs about nuisance parameter, noninformative prior proportional to arbitrary constants is a convenient way to reflect our ignorance for the nuisance parameter. However, such arbitrary constants are improper and unreliable for detection [29]. The information theoretic criteria have been introduced by Akaike [30], Schwartz and Rissanen [31] to compensate the arbitrary resulting penalty function of the criterion. Akaike proposed Akaike information theoretic criteria (AIC), which give the minimum AIC. Schwartz and Rissanen proposed a minimum description length (MDL), which yields the minimum code length. The idea of using the information theoretic criteria with Bernoulli Filter was first presented in [32] for sensor control.

Given a set of *N* observations Z={z(1),⋯,z(N)} and a family of the model defined by PDF $f\left(Z|\hat{\Theta }\right)$, the generalized likelihood function can be written in the form of a penalized logarithmic, given as

(28) $$\mathrm{AIC}=-2\log f\left(Z|\hat{\Theta }\right)+2k\;$$

(29) $$\;\mathrm{MDL}=-\log f\left(Z|\hat{\Theta }\right)+\frac{1}{2}k\log N\;$$

where $\hat{\Theta }$ is the maximum likelihood estimate of the nuisance parameter vector $\Theta =\left\{R_{ss},\sigma^{2}\right\}$, and *k* is the number of free adjusted parameters in Θ. Substituting the MDL criterion, the log-likelihood functions of the source signal and noise components are

(30) $$\;g_{\mathrm{MDL},k}\left(Z_{s,k}|x_{k}\right)=-N\log\left|{\hat{R}}_{ss}\right|-N-\frac{1}{2}\log N\;$$

and

(31) $$\;g_{\mathrm{MDL},k}\left(Z_{n,k}|x_{k}\right)=-N\log\left|{\hat{R}}_{nn}\right|-N\left(M-1\right)-\frac{1}{2}\log N\;$$

Summing up (30) and (31), the total generalized likelihood function based on MDL is given as

(32) $$\;g_{\mathrm{MDL},k}(Z_{k}|x_{k})=-N\log\left(\left|{\hat{R}}_{ss}\right|\left|{\hat{R}}_{nn}\right|\right)-\log N-MN\;$$

Following from the transformation (22), we have

(33) $$\;P_{a\left(\theta_{k}\right)}{\hat{R}}_{k}P_{a\left(\theta_{k}\right)}=G\left(\theta_{k}\right)\left[\begin{array}{cc} {\hat{R}}_{ss} & 0\\ 0 & 0 \end{array}\right]G^{H}\left(\theta_{k}\right)\;$$

where ${\hat{R}}_{k}$ is the sample-covariance matrix given by (6), and also

(34) $$\;P_{a\left(\theta_{k}\right)}^{⊥}{\hat{R}}_{k}P_{a\left(\theta_{k}\right)}^{⊥}=G\left(\theta_{k}\right)\left[\begin{array}{cc} 0 & 0\\ 0 & {\hat{\sigma }}^{2}I_{M-1} \end{array}\right]G^{H}\left(\theta_{k}\right)$$

Taking the trace of both sides, we have

(35) $$\;{\hat{\sigma }}^{2}=\frac{1}{M-1}\mathrm{tr}\left(P_{a\left(\theta_{k}\right)}^{⊥}{\hat{R}}_{k}\right)\;$$

Summing up (33) and (34), taking the determinant of both sides, we have

(36) $$\begin{array}{cc} \left|{\hat{R}}_{ss}\right|\left|{\hat{R}}_{nn}\right| & =\left|P_{a\left(\theta_{k}\right)}{\hat{R}}_{k}P_{a\left(\theta_{k}\right)}+P_{a\left(\theta_{k}\right)}^{⊥}{\hat{R}}_{k}P_{a\left(\theta_{k}\right)}^{⊥}\right|\\ & =\left|P_{a\left(\theta_{k}\right)}{\hat{R}}_{k}P_{a\left(\theta_{k}\right)}+P_{a\left(\theta_{k}\right)}^{⊥}{\hat{\sigma }}^{2}\right|\\ & =\left|P_{a\left(\theta_{k}\right)}{\hat{R}}_{k}P_{a\left(\theta_{k}\right)}+\frac{1}{M-1}P_{a\left(\theta_{k}\right)}^{⊥}\mathrm{tr}\left(P_{a\left(\theta_{k}\right)}^{⊥}{\hat{R}}_{k}\right)\right| \end{array}$$

Substituting into (32) and ignoring the constant *MN*, the log-likelihood function based on MDL can thus be written as

(37) $$\begin{array}{l} g_{\mathrm{MDL},k}\left(Z_{k}|X_{k}\right)\\ =\{\begin{array}{cc} -N\log\left|\frac{1}{M}\mathrm{tr}\left({\hat{R}}_{k}\right)I_{M}\right|-\frac{1}{2}\log N & \mathrm{if}\;X_{k}=\emptyset \;\\ -N\log\left|P_{a\left(\theta_{k}\right)}{\hat{R}}_{k}P_{a\left(\theta_{k}\right)}+\frac{1}{M-1}P_{a\left(\theta_{k}\right)}^{⊥}\mathrm{tr}\left(P_{a\left(\theta_{k}\right)}^{⊥}{\hat{R}}_{k}\right)\right|-\log N & \mathrm{if}\;X_{k}=\left\{x_{k}\right\}\\ 0 & \mathrm{otherwise} \end{array}\; \end{array}$$

where the log-likelihood function conditional upon $X_{k}=\emptyset$ can also be obtained using MDL criterion.

Substituting AIC criterion and separating by a factor of 2, the log-likelihood function based AIC is straightforward, given as

(38) $$\begin{array}{l} \;g_{\mathrm{AIC},k}\left(Z_{k}|X_{k}\right)\\ =\{\begin{array}{cc} -N\log\left|\frac{1}{M}\mathrm{tr}\left({\hat{R}}_{k}\right)I_{M}\right|-1 & \mathrm{if}\;X_{k}=\emptyset \;\\ -N\log\left|P_{a\left(\theta_{k}\right)}{\hat{R}}_{k}P_{a\left(\theta_{k}\right)}+\frac{1}{M-1}P_{a\left(\theta_{k}\right)}^{⊥}\mathrm{tr}\left(P_{a\left(\theta_{k}\right)}^{⊥}{\hat{R}}_{k}\right)\right|-2 & \mathrm{if}\;X_{k}=\left\{x_{k}\right\}\\ 0 & \mathrm{otherwise} \end{array}\; \end{array}$$

By using the log-likelihood function based on either criterion, the variances of source signal and noise can consequently be neglected.

### 3.3. Particle Implementation

The particle filter provides a general framework for the implementation for the Bernoulli filter [21,22]. At time step *k* − 1, the posterior PDF can be approximated by the *q_k_*_ − 1_ and *L* number of weighted particles ${\left\{w_{k-1}^{\left(j\right)},x_{k-1}^{\left(j\right)}\right\}}_{j=1}^{J}$, where $x_{k-1}^{\left(j\right)}=\left[\theta_{k-1}^{\left(j\right)},{\dot{\theta }}_{k-1}^{\left(j\right)}\right]$ is the state vector of particle *j* and $w_{k-1}^{\left(j\right)}$ is its weight. Thus, the posterior PDF is given as:

(39) $$\;s_{k-1}\left(x_{k-1}\right)\approx \overset{J}{\underset{j=1}{\sum }}w_{k-1}^{\left(j\right)}\delta_{x_{k-1}^{\left(j\right)}}\left(x\right)\;$$

where $\delta_{a}\left(x\right)$ is the Dirac delta function concentrated at *a*. Then, the predicted probability of existence *q_k_*_|*k* − 1_ can be computed using Equation (17). The predicted ‘spatial’ PDF $s_{k|k-1}\left(x_{k}\right)$ involves two terms. Assuming the probability of survival, $p_{S,k}\left(x_{k-1}\right)=p_{S}$ is a constant, and the surviving source can be represented by drawing particles *j* = 1, …, *J*, given as

(40) $$\;x_{S,k}^{\left(j\right)}\sim f_{k|k-1}\left(x_{k}|x_{k-1}\right)\;$$

(41) $$\;w_{S,k|k-1}^{\left(j\right)}=\frac{p_{S}\cdot q_{k-1}}{J}\;$$

Assuming the probability of re-entering, $p_{R,k}=p_{R}$ is a constant, and the re-enter source can be represented by drawing particles *j* = 1, …, *B*, given as

(42) $$\;x_{R,k}^{\left(j\right)}\sim f_{R,k}\left(x_{k}\right)\;$$

(43) $$\;w_{R,k|k-1}^{\left(j\right)}=\frac{p_{R}\cdot \left(1-q_{k-1}\right)}{B}\;$$

Since there is no prior knowledge of the re-enter source, the predicted DOA *θ* is a uniform distribution in the state space χ, represents a source that can appear anywhere in the surveillance area. 

After the union of two terms, the predicted ‘spatial’ PDF can be approximated by

(44) $$\;s_{k|k-1}\left(x_{k}\right)\approx \overset{J+B}{\underset{j=1}{\sum }}w_{k|k-1}^{\left(j\right)}\delta_{x_{k}^{\left(j\right)}}\left(x\right)\;$$

Subsequently, the posterior probability of existence *q_k_*_|*k*_ and ‘spatial’ PDF *s_k_*_|*k*_(**x***_k_*) can be obtained using (19) and (20), given as

(45) $$\;q_{k}=\frac{q_{k|k-1}\cdot I_{k}}{\left(1-q_{k|k-1}\right)g_{k}\left(Z_{k}|\emptyset \right)+q_{k|k-1}I_{k}}$$

and

(46) $$\;w_{k}^{\left(j\right)}=\frac{g_{k}\left(Z_{k}|x_{k}^{\left(j\right)}\right)\cdot w_{k|k-1}^{\left(j\right)}}{I_{k}}\;$$

where $I_{k}$ is the integral, given as

(47) $$\;I_{k}=\int g_{k}\left(Z_{k}|x\right)s_{k|k-1}\left(x\right)dx\approx \sum_{j=1}^{J+B}g_{k}\left(Z_{k}|x_{k}^{\left(j\right)}\right)\cdot w_{k|k-1}^{\left(j\right)}\;$$

where $g_{k}\left(Z_{k}|\emptyset \right)$ and $g_{k}\left(Z_{k}|x_{k}^{\left(j\right)}\right)$ are computed by the log-likelihood functions related to Equations (37) or (38) based on either MDL (MDL-TBD-Ber) or AIC (AIC-TBD-Ber) criterion. 

Considering a low SNR and small number of snapshots scenarios, the mainlobe of the distribution of likelihood function is usually flat and spread. In order to enhance the high-likelihood area, we exponentially weight the penalised log-likelihood function using a constant value *r* to enhance performance, given as

(48) $$\;g_{k}\left(Z_{k}|X^{\left(j\right)}\right)={\left(g_{k}\left(Z_{k}|X^{\left(j\right)}\right)-\underset{X^{\left(j\right)}}{\min}g_{k}\left(Z_{k}|X^{\left(j\right)}\right)\right)}^{r}\;$$

for all $X^{\left(0\right)}=\emptyset$ and $X^{\left(j\right)}={\left\{x^{\left(j\right)}\right\}}_{j=1}^{J+B}$. Thus, the likelihood function becomes more peaky and amenable to our problem. The choice of weighting factor r can be determined based on simulations in Section 4. Finally, the particles are resampled *J* times and weights are equals to 1/*J*. If the posterior probability of existence follows *q_k_* > 0.5, a source exists, and the corresponding posterior DOA is equal to

(49) $$\;{\hat{\theta }}_{k}=\overset{J}{\underset{j=1}{\sum }}\theta_{k}^{\left(j\right)}\;$$

The pseudo-code of the proposed BPF is presented in Algorithm 1.

**Algorithm 1.** Bernoulli filter for detection and DOA trackingInput: $q_{k-1},\;{\left\{w_{k-1}^{\left(j\right)},x_{k-1}^{\left(j\right)}\right\}}_{j=1}^{J}$,1: Compute $q_{k|k-1}$ using (17)2: Draw surviving source particles: $x_{S,k}^{\left(j\right)}\sim f_{k|k-1}\left(x_{k}|x_{k-1}\right)$ with weights $w_{S,k|k-1}^{\left(j\right)}$ for *j* = 1, …, *J*3: Draw re-entering source particles: $x_{R,k}^{\left(j\right)}\sim f_{R,k}\left(x_{k}\right)$ with weights $w_{R,k|k-1}^{\left(j\right)}$ for *j* = 1, …, *B*4: Union of weighted particles: ${\left\{w_{k|k-1}^{\left(j\right)},x_{k}^{\left(j\right)}\right\}}_{j=1}^{J+B}={\left\{w_{S,k|k-1}^{\left(j\right)},x_{S,k}^{\left(j\right)}\right\}}_{j=1}^{J}\cup {\left\{w_{R,k|k-1}^{\left(j\right)},x_{R,k}^{\left(j\right)}\right\}}_{j=1}^{B}$5: Compute likelihood function according to (37) or (38) for *j* = 1, …, *J* + *B*6: Normalize and exponential likelihood function using (48)7: Compute *q_k_* and $w_{k}^{\left(j\right)}$ using (45) and (46)8: Resample *J* times to obtain *J* particlesOutput: $q_{k},\;{\left\{w_{k}^{\left(j\right)},x_{k}^{\left(j\right)}\right\}}_{j=1}^{J}$

## 4. Discussions

### 4.1. Performance Metric

In order to evaluate the detection and DOA tracking performance simultaneously, a metric should be chosen that not only computes the error between the estimated and true angles, but also can quantify penalties when a source that is present is missed or a source that does not exist is falsely detected. In this section, we use the optimal sub-pattern assignment (OSPA) error metric [33] to evaluate performances. A penalty value is employed in OSPA to transfer the cardinality error into the state error and then OSPA is able to present the performance on source number estimation as well as source DOA estimation.

For joint detection and DOA tracking, the construction of the OSPA distance $d_{\mathrm{OSPA}}^{\left(c\right)}\left(X,Y\right)$ between two finite sets *X* and *Y* with a cardinality of at most one is as follows

(50) $$\;d_{\mathrm{OSPA}}^{\left(c\right)}\left(\emptyset ,\emptyset \right)=0$$

(51) $$\;d_{\mathrm{OSPA}}^{\left(c\right)}\left(\left\{x\right\},\emptyset \right)=d_{\mathrm{OSPA}}^{\left(c\right)}\left(\emptyset ,\left\{y\right\}\right)=c$$

(52) $$d_{\mathrm{OSPA}}^{\left(c\right)}\left(\left\{x\right\},\left\{y\right\}\right)=\min\left(\|x-y\|,c\right)$$

The cut-off parameter c determines the relative weighting of the penalties assigned to cardinality and localization errors and a moderate cut-off value c=10 will be employed in our paper. 

### 4.2. Computational Complexity

The complexity of TBD-Ber filters are generally similar to that of the standard particle filter which can be found in [34,35]. Each particle is drawn, updated and resampled, in a similar manner. The main difference lies in the computation of the likelihood for each particle and the existence probability. From (37) and (38), the computation of the likelihood requires $O\left(\left(J+B+1\right)M^{2}\right)$ evaluations of the particles. 

For comparison, we choose the following classical array signal processing methods. Firstly, the MVDR method is selected due to its popularity and simplicity. According to the MVDR criterion, a spatial spectrum is generated and a set of detectors is obtained by picking peaks above a selected threshold. Since it relies on estimating and inverting a covariance matrix, it requires $O\left(M^{3}\right)$. Intuitively, the computation load severely increases when the number of elements M increases. Comparatively, grid searching is avoided for the TBD-Ber filters by drawing state samples randomly over the state-space.

Secondly, a maximum likelihood (ML) estimator coinciding with AIC or MDL criteria is selected. More specifically, it requires computing ML estimates for a series of nested models and selecting the one that best fits the underlying criteria. Usually, an alternating maximization (AM) technique is employed to transform such multi-dimensional optimization problems into a sequence of much simpler one-dimensional optimization problems that are iterated until convergence. However, it still has high computational complexity since it requires ML estimates for all candidate models.

### 4.3. Weighting Factor r

**Figure 1:**
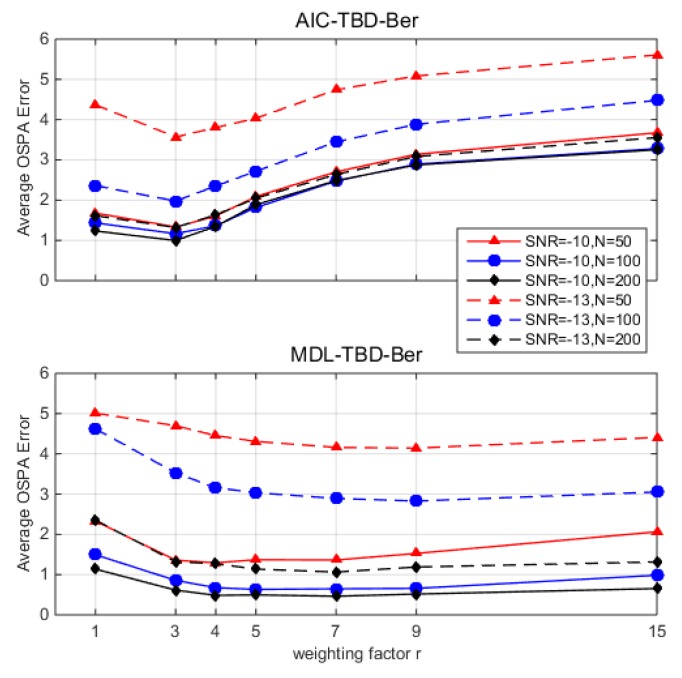
Optimal sub-pattern assignment (OSPA) error averaged over time steps (100 Monte Carlo runs) versus different weighting factors *r* = [1, 3, 4, 5, 7, 9, 15]. Akaike information criterion track-before-detect Bernoulli filter (AIC-TBD-Ber); minimum description length track-before-detect Bernoulli filter (MDL-TBD-Ber); signal-to-noise ratio (SNR).

In our algorithm, the likelihood function is enhanced by exponentially weighting via a factor *r* in (26). Figure 1 shows the average OSPA error for TBD-Ber approaches (AIC-TBD-Ber and MDL-TBD-Ber) versus different weighting factors *r* = [1, 3, 4, 5, 7, 9, 15] over 100 Monte Carlo runs. The detailed simulation setup is provided in Section 5. As we can see, both TBD-Ber approaches have better performances when *r* is set around 3. It is worth mentioning that slightly larger or smaller than 3 will not lead to a significant difference. 

## 5. Simulations

In this section, several simulations are organized to investigate the performance of the proposed algorithm developed in this paper. Considering an unknown and time-varying number of sources, the performance is demonstrated by a single run and a Monte Carlo simulation in different experimental environments. Note that the RJMCMC method [10] can only be carried out using a single snapshot and completely fails in low SNR. In order to make a fair comparison with the proposed method which tracks the DOAs in time rather than just detect in each time step, we post-process the detections of MVDR and AIC/MDL based ML estimator using a standard Bernoulli-particle filter [21], resulting in MVDR-TAD-Ber, AIC-TAD-Ber, and MDL-TAD-Ber.

The array is a standard uniformly linear array with a spacing of d=1.5 m using M=6 sensors. A single narrowband source signal is generated with frequency f=500 Hz. Noise variance $\sigma_{n}^{2}=1$ is assumed to be unknown and fixed. SNR is computed as $10\log\left(\sigma_{s}^{2}/\sigma_{n}^{2}\right)$. The exponentially weighting factor is r=5 which has been studied in Section 4.3. The following simulations assume there is no source present from time step 1 to time step 15, one source appears from time step 16 to time step 40 and disappears from time step 41 to time step 50. The initial DOA of the source is θ=−30°. The DOA velocity is around $\dot{\theta }=2{}^{\circ}/s$ with variance $\sigma_{\theta }^{2}=0.1$.

The parameters for the TBD-Ber approaches are as follows: number of particles J=1000, B=200 and $p_{S}=0.95$, $p_{R}=0.05$. Since we have no prior of the source, the initial DOA is assumed to be uniformly distributed over $\theta_{0}\sim U\left(-90,90\right]$, and the initial probability of existence is $q_{0}=0.5$. 

The parameters for the TAD-Ber approaches are as follows: number of particles J=1000, B=200 and $p_{S}=0.95$ $p_{R}=0.05$, the probability of detection is $p_{D}=0.6$ due to low SNR simulations and the variance of the measurement noise equal to 1. The birth model is also a uniform distribution from −90° to 90°. 

The MVDR-TAD-Ber detection threshold is a selected factor times the median MVDR power over DOA. The searching step in the pre-processing module of TAD-Ber approaches is 0.5°. It is worth mentioning that it is not easy to determine the clutter rate (number of false detectors) in practical applications. We simulated several times to find a better choice of clutter rate: MVDR-TAD-Ber equals 0.1, AIC-TAD-Ber equals 1.5, and MDL-TAD-Ber equals 0.8. 

### 5.1. Single Runs

**Figure 2:**
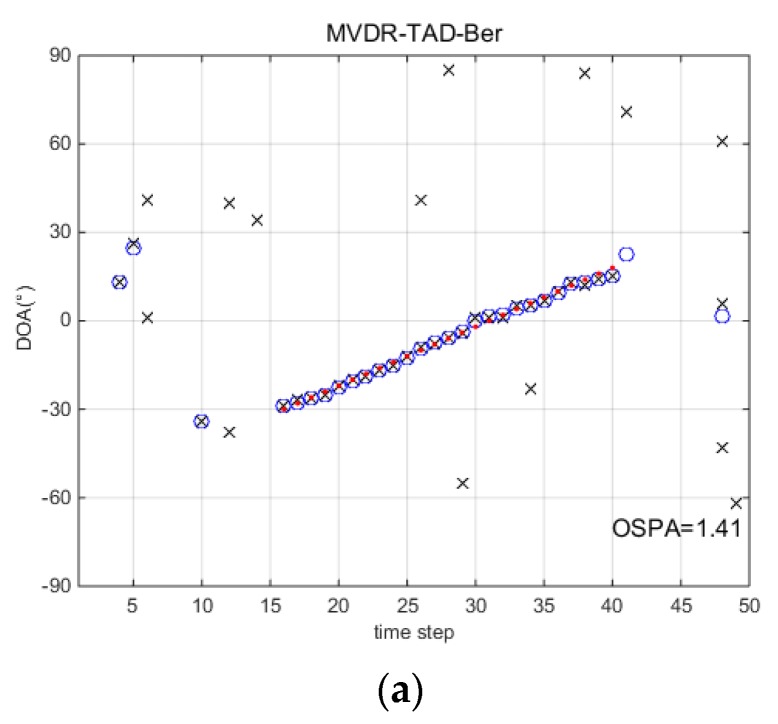

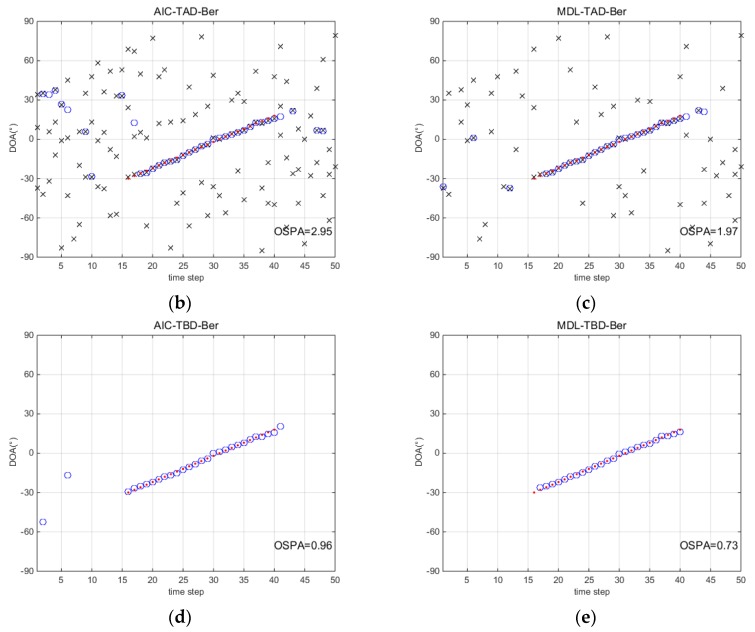
Direction-of-arrival (DOA) tracking results with signal to noise ratio (SNR)=−8 dB and snapshots N=50. (**a**) minimum-variance-distortionless-response (MVDR)-TAD-Ber; (**b**) AIC-track-after-detect (TAD)-Ber; (**c**) MDL-TAD-Ber; (**d**) AIC-TBD-Ber; (**e**) MDL-TBD-Ber.

**Figure 3:**
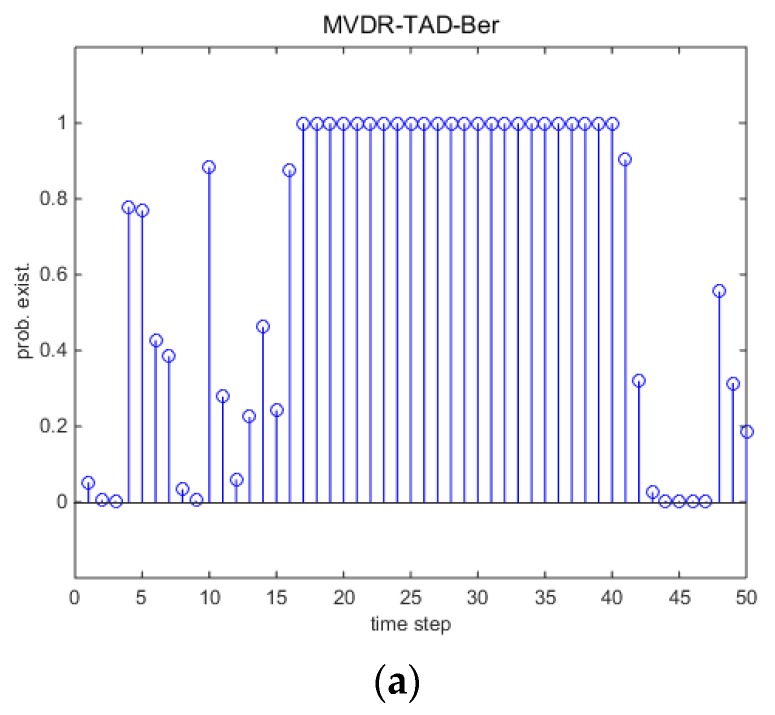

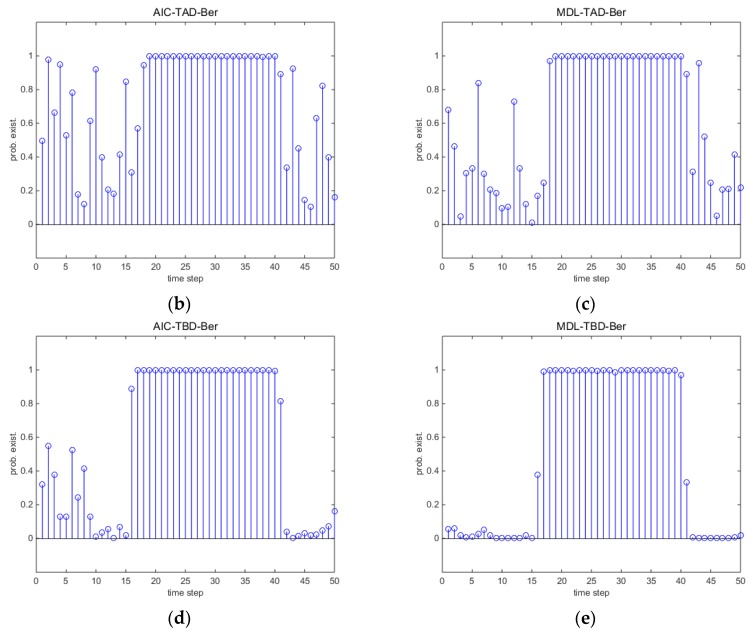
Detection results with SNR=−8 dB and snapshots N=50. (**a**) MVDR-TAD-Ber; (**b**) AIC-TAD-Ber; (**c**) MDL-TAD-Ber; (**d**) AIC-TBD-Ber; (**e**) MDL-TBD-Ber.

This section gives the detection and tracking results by one trail comparing with different methods. Figure 2 presents the DOA tracking results with SNR=−8 dB and snapshots N=50. Red dots are the true source localizations and the blue circles are the tracking results. Black ‘x’s are the detections of the TAD-Ber filters which correspond to the input of a standard BPF. Intuitively, without tracking procedure, the black ‘x’s are unsatisfactory by using classical array signal processing algorithms. Although the clutter rate is carefully selected, the performance of TAD-Ber filters are still affected by the false detectors as a consequence. For the TBD-Ber filters, they not only reduce the false detectors, but also are able to detect and localize the source correctly when a source exists. It is more clear in Figure 3d,e that the probability of source existence $q_{k}$ remains low when the source does not exist. After 16th step, $q_{k}$ rapidly rises close to 1.0 when the source appears and remains close to 1.0 to time step 40.

**Figure 4:**
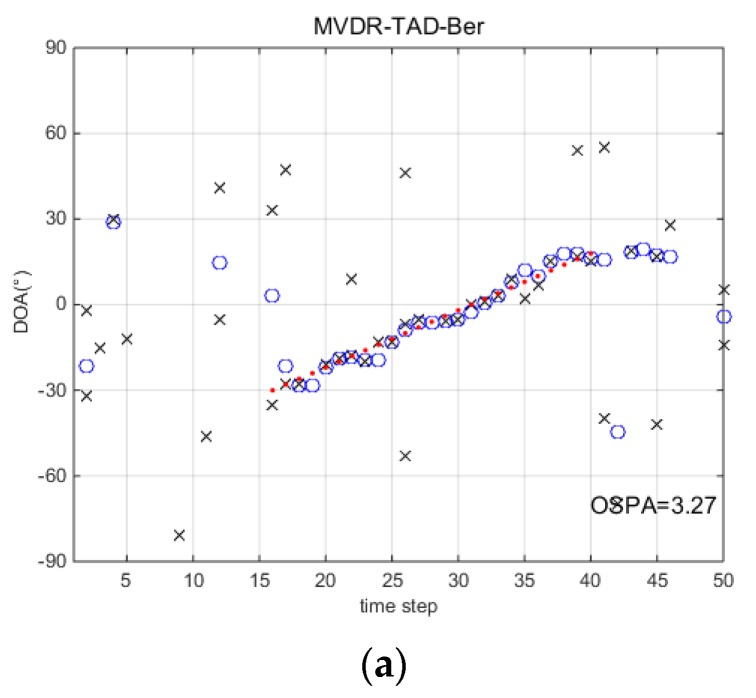

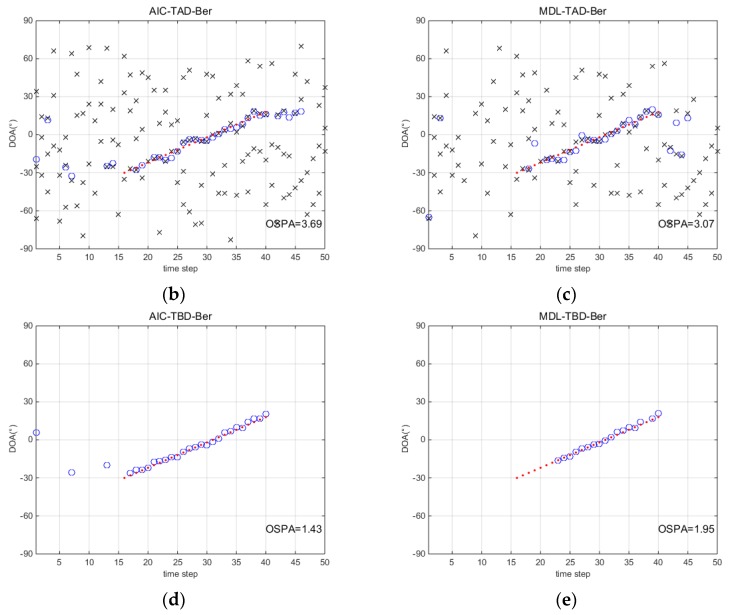
DOA tracking results with SNR=−14 dB and snapshots N=200. (**a**) MVDR-TAD-Ber; (**b**) AIC-TAD-Ber; (**c**) MDL-TAD-Ber; (**d**) AIC-TBD-Ber; (**e**) MDL-TBD-Ber.

**Figure 5:**
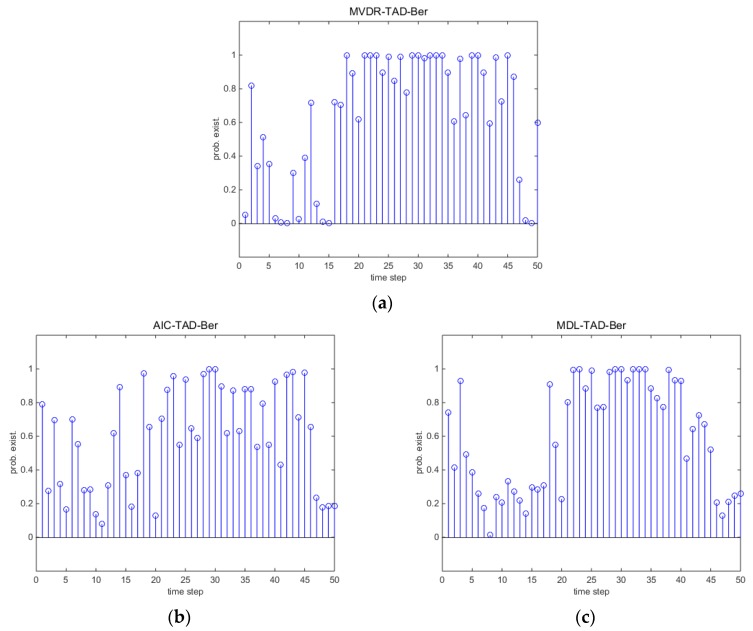

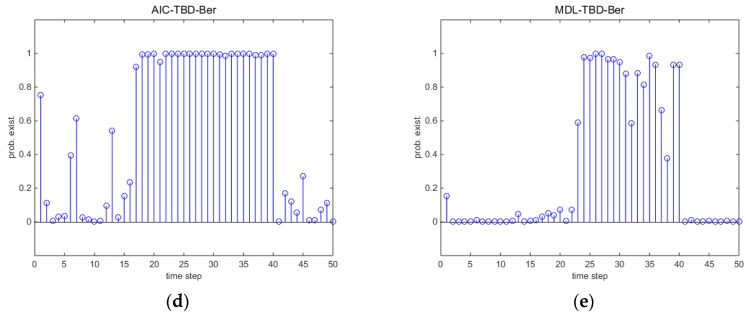
Detection results with SNR=−14 dB and snapshots N=200. (**a**) MVDR-TAD-Ber; (**b**) AIC-TAD-Ber; (**c**) MDL-TAD-Ber; (**d**) AIC-TBD-Ber; (**e**) MDL-TBD-Ber.

Figure 4 and Figure 5 consider a more severe environment where SNR=−14 dB and snapshots N=200. Accompanied with the decrease of SNR, the measurements are seriously distorted by the noise. Intuitively, the performance of TAD-Ber filters is seriously affected by the noise environment. MDL-TBD-Ber suffers from underestimation, and hence miss detects many points at the start of the trajectory. However, AIC-TBD-Ber is able to localize the DOA accurately and consistently lock on the trajectory when source exist. The better performance of $q_{k}$ can be seen in Figure 5. 

### 5.2. Monte Carlo Runs

**Figure 6:**
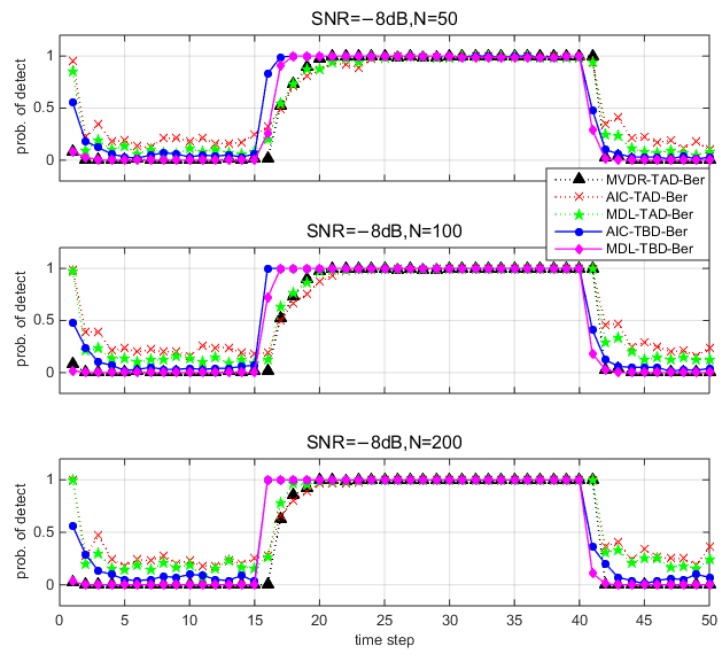
Detection performance for different methods (100 Monte Carlo runs) when SNR=−8 dB and number of snapshots N=50, 100, 200.

**Figure 7:**
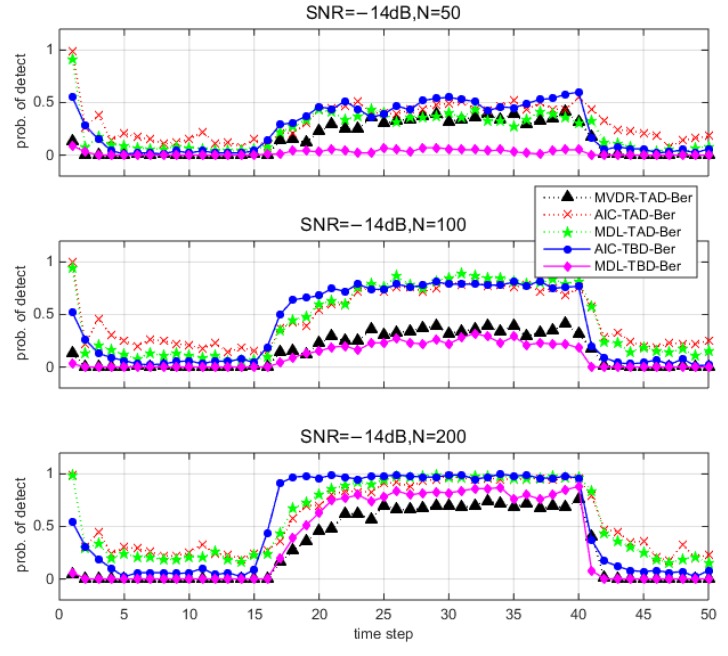
Detection performance for different methods (100 Monte Carlo runs) when SNR=−14 dB and number of snapshots N=50, 100, 200.

This section presents the average performance via Monte Carlo simulations. Figure 6 and Figure 7 show the detection results for different methods under different environments. Note that the y-axis denotes the probability of detection among 100 trails. Since there is no source from time step 1 to 15 and time step 41 to 50, the y-axis denotes the probability of false alarm $p_{F}$. Since the source exists from time step 16 to 40, the y-axis denotes the probability of correct detection $p_{D}$. TBD-Ber filters reduce the probability of false alarm $p_{F}$ compared to TAD-Ber filters. MDL-TBD-Ber performance is affected by underestimation errors under low SNR and small number of snapshots, and thus results in an attendant loss in detection sensitivity ($p_{D}$ is very low). AIC-TBD-Ber demonstrates better performance in all simulated scenarios.

**Figure 8:**
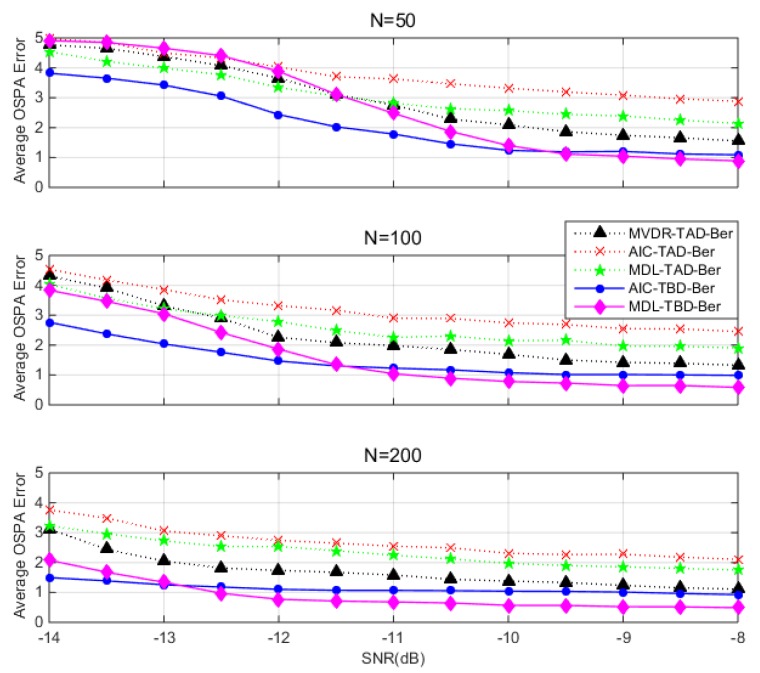
OPSA error averaged over time steps (100 Monte Carlo runs) versus SNR. Vertical panels (top to bottom) vary in number of snapshots N=50, 100, 200.

Figure 8 shows the average OSPA error versus different SNR via 100 trails. OSPA errors are averaged over time steps and 100 trails. It presents the superior performance of TBD-Ber filters compared to TAD-Ber filters. In the high SNR region, MVDR-TAD-Ber show a comparable performance when SNR is 8 dB since the detections become so accurate that the accuracy could catch up with TBD-Ber filters. MDL-TBD-Ber gives better performance when SNR>−10 dB. The performance of AIC-TBD-Ber is affected by overestimation errors and slightly worse than MDL-TBD-Ber. In the low SNR region, the performance of MDL-TBD-Ber degrades rapidly due to low SNR and small snapshots, while AIC-TBD-Ber retains better performance compared to other filters. Simulations verify the proposed track-before-detect Bernoulli filter is a more robust detection and tracking of a single source in comparison if the TAD approaches especially in noisy condition.

The comparison of computational time for 50 time steps over 100 Monte Carlo simulations is given in Table 1. The simulations are carried out on a computer with an i5 processor and 8 GB of RAM, and all programs were coded and run in MATLAB. 

## 6. Conclusions

In this paper, the authors focus on solving the problem of joint detection and DOA tracking using an array of sensors. For this problem, we propose the use of measurements obtained from array elements’ raw data. We implemented the Bernoulli filter, which emerged from the RFS framework. Since more information is reserved, the performance of detection and DOA tracking is improved by using these TBD measurements. The performances of TBD-Ber and TAD-Ber approaches are evaluated based on simulations which verify the robustness of the proposed TBD-Ber filters compared to TAD-Ber filters.

Our future work will focus on more complicated situations, such as propagation path loss, or a moving source that might move to near-field from the array, etc.

## Figures and Tables

**Table 1:** Computation time of different algorithms per iteration.

Algorithm	Mean Duration ± Standard Deviation
MVDR-TAD-Ber	0.078±0.0002
AIC-TBD-Ber	0.037±0.0010
MDL-TBD-Ber	0.042±0.0014
AIC-TAD-Ber	0.102±0.0023
MDL-TAD-Ber	0.094±0.0018
